# Use of Digital Technology as a Collaborative Tool among Nursing Students—Survey Study and Validation

**DOI:** 10.3390/ijerph192114267

**Published:** 2022-11-01

**Authors:** Natalia Fischer-Suárez, David Lozano-Paniagua, Jessica García-González, Gracia Castro-Luna, Mar Requena-Mullor, Raquel Alarcón-Rodríguez, Tesifón Parrón-Carreño, Bruno José Nievas-Soriano

**Affiliations:** 1 Department of Nursing, Physiotherapy, and Medicine, University of Almería, 04120 Almeria, Spain; 2 Department of Obstetrics and Gynecology, Torrecárdenas Universitary Hospital, 04009 Almeria, Spain; 3 Andalusian Council of Health and Families at Almería Province, 04005 Almeria, Spain

**Keywords:** digital technology, eHealth, health technology, questionnaire, nursing, validation

## Abstract

Background: This research aimed to develop a questionnaire to analyze perceived aspects of using digital technology among nursing students as a collaborative tool. We further sought to evaluate the psychometric reliability of the instrument. Methods: A cross-sectional observational study was performed using a questionnaire developed from scratch. Psychometric studies and univariate and bivariate analyses were performed. Results: 132 nursing students participated. The exploratory and confirmatory analyses of the questionnaire excluded 4 of the initial 18 items and established four domains, and internal consistency was found. The mean global score of the answers to the questionnaire was 4.67 on a scale of 1–5 points, and all the domains obtained high scores. Men scored higher on the usefulness and the global score, while no differences were found regarding age. Conclusions: Nursing students positively assess the use of digital technology as a collaborative tool, regardless of age. Digital technology as a collaborative tool is perceived as beneficial, improves their involvement, and allows nursing students to obtain a better knowledge of their partners. These findings can help develop group projects and tools based on technology to train future nursing professionals. The questionnaire developed is a valid tool to assess this.

## 1. Introduction

Digital technology has become integral to nurses’ practice [1,2], and nursing students’ predispositions toward technology can affect their future use of technology in clinical settings [3]. Nursing students have a unique perspective on how digital technologies can support learning in clinical practice [4]. Digital technology provides new opportunities to support teaching and learning [5]. Incorporating digital tools to help learn is essential to support the delivery of content and the use of technology by students [2]. Thus, it is necessary to prepare future generations of nurses to be active participants in information and communications technology, digital health, and eHealth rather than just teaching them technical skills regarding these tools [6].

Digital competence is vital for involving professional knowledge and skills to facilitate the educational use of digital technologies [5], and educators must pay attention to the training and education of nurses [7]. Integrating digital technologies into the nursing curriculum for students is necessary, and nursing schools must incorporate digital technology into the teaching environment. Nurse educators can support the integration of digital technology into pedagogy [2], as creative digital-based approaches can help engage students and offer advantages, such as convenience, ease of administration, and analysis [8]. Technology-based activities that encourage playability may promote learning among nursing students [9,10].

Digital technologies add value to learning because students feel comfortable with digital learning and face-to-face interaction activities. The students easily integrate digital technologies into learning, which helps learning and social fellowship. Thus, new forms of education based on technology and physical interaction with partners should be experienced [5,6]. Nursing associations recommend integrating information technology into nursing curriculums, and nurse educators have begun to address technology integration in the nursing curriculum [11]. As other authors state, while recent work from the field of mobile learning has emphasized the importance of learning across contexts, little analytical attention has been paid to the underlying dynamics of this phenomenon [12].

Nevertheless, not many nursing educators have studied the results of combining digital technologies and collaborative work with face-to-face interaction [5,6]. Some authors have stated that using innovative educational tools by nursing educators can be daunting because of the steep learning curve [13]. Consequently, nursing curricula are outdated in many countries, as there are considerable gaps in technology accessibility and training in nurse education. Another challenge is obtaining a better understanding of how digital technology impacts the well-being of students [14]. The effectiveness of learning among undergraduate nursing students in subjects such as anatomy or physiology can be higher when digital methods or problem-solving capacities are applied, provided these techniques facilitate students’ enjoyment of learning [15,16].

Digital technologies are already benefiting nursing practice and education. Digital programs in which nurses provide daily monitoring have helped reduce emergency department admissions. Mobile devices enable nurses to complement nursing education by providing innovative pedagogical solutions for content delivery and distance learning opportunities [17]. In nursing education, digital technology is used in the clinic and classroom to supplement learning. However, despite its upward trend, there is still a gap in acceptance [18]. The use of digital educational technologies is still at an early stage. Both students and teachers are still unaware of the scope and possibilities of these tools and do not take advantage of the full potential they can offer. The full impact of these technologies on personalized nursing care learning is not yet known [19]. Nowadays, we all carry electronic devices in our pockets. We must learn to integrate them into the working life of professionals and the academic life of nursing students.

Henceforth, there is a need to explore how digital educational technologies can be a part of learning in nursing education [5] without forgetting the importance of the humanistic approach and face-to-face interaction among peers [5,6]. Although many nursing programs have tried to implement digital technology in the classroom, more research in this field must be conducted [11]. Little is known about students’ experience using digital technology to learn about health [20,21], and reports of formal education using technology applications are low [3]. The ultimate goal of technology as a collaborative tool in healthcare practice is to improve the collaborative group as, in the future, nursing students will work in collaborative teams. As described by other authors, it is essential to estimate the validity and reliability of assessment tools in this study [16]. Nevertheless, we did not find a validated questionnaire to evaluate this specific aspect. Thus, we decided to perform a study to analyze the perceived usefulness of digital technology as a collaborative tool in a health project conducted by students at the University of Almeria, Spain, nursing degree. Our guiding research question was: what is the perceived usefulness of using digital technology among nursing students in a collaborative health project?

Therefore, the main aim of this research was to analyze the perceived usefulness of using digital technology among nursing students in a collaborative health project. We further sought to develop a questionnaire to perform this task and evaluate its psychometric reliability, performing exploratory and confirmatory factorial analyses.

## 2. Materials and Methods

### 2.1. Study Design

A cross-sectional observational study was performed through a questionnaire to evaluate digital technology’s perceived usefulness in creating a group project with peers. The students’ project aimed to develop a digital instrument in small groups of three to six students that taught anatomy to their partners entertainingly using digital technology as a collaborative instrument. Two examples of the projects developed are included as supplementary materials.

The study consisted of two phases: first, the development and validation of the questionnaire; second, the analysis of the data obtained from the participants’ answers. The questionnaire to assess the project’s perceived usefulness was developed based on three aspects: first, a literature review, performed using the terms questionnaire, digital technology, and nursing students in databases such as Pubmed, Scopus, and Web of Science; second, the professional and college experience of the authors of this study; third, meetings with the students to retrieve information. In the meetings, the students were asked to express their concerns about the difficulties they had in understanding the signature and how they thought it could improve, allowing us to develop the initial items of the questionnaire. The results of the meetings were merged with the literature reviewed. Thus, every questionnaire item corresponded to every concern detected in the face-to-face interviews. The initial version contained 20 items: 2 were demographical items and 18 were evaluation items (Table 1). Responses for the evaluation items were five-point Likert scales. Possible scores for the 18 qualitative items were the following: 1 = not at all, 2 = a little, 3 = somewhat, 4 = quite a bit, 5 = a lot. Reliability and validation analyses were performed [22].

### 2.2. Sample Size and Pilot Testing

The Epi Info™ App (Centers for Disease Control and Prevention, Atlanta, Georgia), Version 16 November 2021, was used to estimate the sample size. The population size was 47,229 (number of nursing students in Spain) [23], and the confidence interval was 95%, with an expected frequency of 91% and a precision of estimate within 5% of either side of the actual population proportion. From these values, a required sample size of 126 participants was established. It was in line with the classic rule set by Kline et al. [24] of using 2 to 20 subjects for each questionnaire item.

The eligible population was nursing students enrolled in the course of human anatomy from the Health Sciences Faculty of the University of Almeria, Spain. This program was chosen as the subject of human anatomy is one of the most challenging subjects for students, with a high failure rate among first-year students, so it is susceptible to the application of innovative methodologies that facilitate learning. Four students who did not participate in the research evaluated the feasibility, simplicity, and time required to answer the questionnaire. After concluding the exposition of the collaborative health projects, a printed version of the questionnaire was given to the nursing students who voluntarily took part in the study. The initial text of the questionnaire showed the purpose of the questionnaire and the informed consent. Personal data were not collected, and the participants could withdraw anytime. Only fully completed questionnaires were accepted. A database was created with all the information collected from statistical analyses without any statistical correction.

### 2.3. Questionnaire Validation

An expert panel composed of five nursing professionals and college professors performed content validation. The adequacy of the exploratory factor analysis (EFA) was determined using Bartlett’s test and the Kaiser–Meyer–Olkin (KMO) measure. For the evaluation of construct validity, the 18 qualitative items of the questionnaire were assessed through exploratory factor analysis. For the study of the item structure, principal factors calculation with a Varimax rotation was used. The decision to include an item in a construct was made iteratively by checking factor loadings and Cronbach’s alpha to determine which items did not sufficiently measure the same underlying construct [25]. For the inclusion of a question in a construct, items with a Pearson correlation coefficient lower than 0.5 were discarded. The latent dimensions formed by the items were determined.

Confirmatory factor analysis (CFA), which is the most used method in the literature [26] and has been used in similar research [27], was completed using AMOS software’s maximum likelihood estimation method. The goodness of fit was evaluated using the most common fit indices used in the literature: the chi-square test, the goodness of fit index (GFI), the root mean square error of approximation (RMSEA), the normed fit index (NFI), the non-normed fit index (NNFI) or the Tucker–Lewis index (TLI), and the comparative fit index (CFI) [22]. These values, which can range from 0 to 1, indicate a good model fit the closer they are to the value 1. In the case of the RMSEA, its value should be below 0.08. The reliability of the questionnaire was assessed through its internal consistency using Cronbach’s alpha [28], the split-half method, Guttman’s lambda test, parallel and strictly parallel models, and the intraclass correlation coefficient (ICC). In the case of these tests, whose results can range from 0 to 1, the closer the results are to the value 1, the higher the reliability.

### 2.4. Statistical Analyses and Review Board Approval

The data were analyzed using the SPSS version 27 (IBM Inc., Armonk, NY, USA). The nonparametric Mann–Whitney U test for independent variables was used to investigate the effect of sex. The Kolmogorov–Smirnov test was used for testing normality. Spearman’s rho correlation coefficient was also applied to analyze the existence of a linear association between domain scores and age. The statistical software AMOS version 26.0.0 (IBM Inc., Armonk, NY, USA) was used for confirmatory factor analysis. All methods described in this study were endorsed by the Research and Ethics Committee of Nursing, Physiotherapy, and Medicine Department of the University of Almeria (Spain), with approval number 178/2022. The questionnaire did not gather personal information. All participants voluntarily agreed to participate in the study. Informed consent was shown at the beginning of the questionnaire.

## 3. Results

### 3.1. Demographic Characteristics of the Respondents

A total of 132 nursing students took part in the research. Since the population study consisted of 136 nursing students, the participation rate was 97%. The attrition rate was 0%. The mean age of the respondents was 21.3 years, with a standard deviation (S.D.) of 6.8 years. The median was 18.5 years (Table 2).

### 3.2. Questionnaire Validation

Regarding the evaluation by the expert panel, the content validity index was 1.00 for the questionnaire, on a scale of 0 to 1, with 1 being the best possible value. The KMO value was 0.843, and the result of Bartlett’s test was *p* < 0.001, which indicated adequacy to perform exploratory factorial analysis. The exploratory factor analysis excluded 4 of the 18 initial items from the questionnaire and established four domains, which explained 63.9% of the variance in the data (Table 3). Domain one was measured by seven items, domain two by three items, domain three by two items, and domain four by two (Table 4). The domains were named based on the items included: the first domain defined the usefulness of the students’ projects, the second domain defined the involvement of the partners, the third domain defined the usefulness of the other students’ projects, and the fourth domain described better knowledge of partners.

The global Cronbach’s alpha for the questionnaire was 0.85. The results of the other reliability tests stood between 0.783 and 0.879 (Table 5).

**Figure 1:**
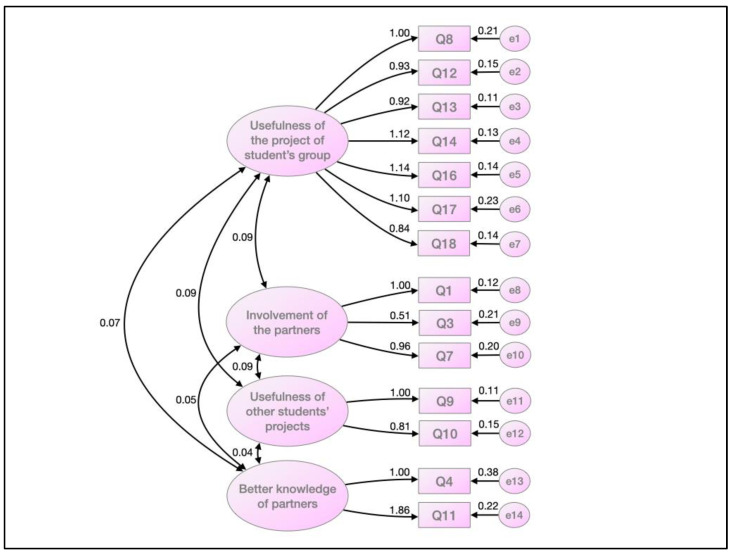
Measures of internal consistency of the construct by confirmatory factor analysis, applying maximum likelihood estimation method.

Confirmatory factor analysis was performed to assess the validity of the model generated through the exploratory factor analysis. The CFA was performed by applying the maximum likelihood estimation method. The model revealed 71 degrees of freedom, with a Chi-square value of 109.093 and a probability level of *p* < 0.005. The construct’s internal consistency is shown in Figure 1. The four constructs observed in EFC were confirmed, with the items exhibiting correlations from 0.51 to 1.14.

Regression values, critical ratio, standard errors, and significance were estimated (Table 6). Critical ratio weights were high, and the disparities were significant in all the parameters.

The model’s goodness of fit was estimated through the following indices (Table 7): The GFI value was 0.898. The value of the magnitude evaluation of the RMSEA was 0.064. The NFI was 0.839, the NNFI (or TLI) value was 0.917, and the CFI value was 0.935.

### 3.3. Univariant Analysis

The analysis of the score of the domains adjusted by the number of items of each domain showed that, on a scale of 1–5 points, five being the best possible rating, the mean global score of the questionnaire was 4.67 (Table 8). The domain with the lowest score was the better knowledge of the partners, with 4.48 points. The domain with the highest score was the usefulness of other students’ projects, with a score of 4.75.

### 3.4. Bivariant Analysis

The analysis of the scores of the domains regarding sex (Table 9) showed that men scored higher in the domain that defined the project’s usefulness of the student group and the global score of the activity. No differences were found in the rest of the domains.

The analysis of the domains’ scores regarding the participants’ age (Table 10) did not find any correlation between either aspects.

## 4. Discussion

The primary purpose of this study was to create a questionnaire to assess the perceived usefulness of building a collaborative health project using technology among nursing students. We also aimed to evaluate the psychometric reliability of the instrument. As far as we know, this is the first investigation to create and test a questionnaire designed with this specific aim. Making an assessment tool is not straightforward [22], as objective tests must be applied to ensure validity. From the students’ perspective, the results from the evaluation could be helpful for the clinical practice of nursing professionals and the research of learning tools and models based on technology.

### 4.1. Size and Composition of the Sample

While some authors recommend sample sizes between 50 and 100 participants to conduct factor analysis and evaluate the psychometric properties of questionnaires [22], other authors propose applying the rule of Kline [24]. Others advise using tools such as Epi Info™ [29]. This tool calculated a needed sample size of 126 participants in our case, which agreed with the figures stated by other authors [24,30,31]. We gathered 132 questionnaires, above the figures suggested by other authors [22,24]. The response rate received was also higher than the figures stated in other studies [5,7]. Our sample was a convenience sample of nursing students, with a mean of 18.5 years. Similar to in other studies with nursing students, most were women [32], who tend to use more digital technology for health aspects than men [33]. This aspect must be considered when evaluating the external validity of the evaluation results.

### 4.2. Questionnaire Validation

The exploratory factor analysis led us to draw four items from the initial 18-item questionnaire. This number of items concurs with the figures expressed by other authors [26]. The exploratory factor analysis of the consequent 14-item questionnaire detected consistency in four domains. These domains were analyzed based on what the items shared [17]. The factorial solution demonstrated the model, similar to other questionnaires assessed in other ambits [7,22,34,35]. Thus, the theoretical construct of the questionnaire was adequate. Each questionnaire domain defined a different dimension, not related to the other domains, but well defined by the questions that compose it.

Regarding reliability, Cronbach’s alpha coefficient is the technique most employed in the literature [22]. Thus, it was used to assess the internal consistency of the questionnaire. An Alpha value higher than 0.67 could be considered acceptable [35], but Alpha values more elevated than 0.80 are regarded as good [22] or excellent [34]. In our resulting 14-item questionnaire, Cronbach’s alpha value was 0.85, exceeding the figures displayed by other authors in similar research [31]. The Intraclass correlation coefficient demonstrated high reliability, with the lower and upper scores being higher than other figures [31]. The remnant reliability tests also scored high values.

The confirmatory factorial analysis is considered adequate to define a questionnaire’s underlying conceptual structure [36], and in our case, the sample size was beyond the figures suggested in the literature [22,24]. Regarding the evaluation of RMSEA, weights under 0.08 indicate a good fit, which was our case. NFI values beyond 0.9 are considered good, and this was our case. TLI values above 0.9 are believed optimal, as was our case. Therefore, the CFI and the TLI are within the preferred range in our final questionnaire. The NFI and GFI values are above the minimum weight, and the RMSEA is within the optimal range. Therefore, the previously defined domains were verified.

### 4.3. Univariant Analysis

All the domains of the questionnaire obtained high scores. It implies that their project and the other students’ projects were helpful from the student’s point of view. The partners were involved in the project, and the activity benefited from understanding the partners better; therefore, the overall evaluation of using technology for collaborative research was positive. A possible explanation for these high scores is that using technology-based activities that encourage playability may promote learning among nursing students [9,10]. Nevertheless, we must consider a possible positive overestimation due to the potential selection bias of our sample and the volunteer effect [37]. In this sense, other authors state [8] that the potential advantages of using innovative approaches similar to this could outweigh the problems associated with the potential and unavoidable selection bias.

### 4.4. Bivariant Analysis

Regarding sex, most nursing students in Spain are women [32] who use more digital technology for health aspects than men [33]. Our sample was indeed mainly composed of women. Nevertheless, the male students scored higher on one of the domains, the project’s usefulness, and the activity’s global score. A plausible explanation is that female nursing students may be more used to using technology in health, so they could have been less surprised by the advantages of using technology to perform the activity [38].

The age of our research participants was mainly similar to other research related to the use of technology among nursing students [9,39]. When analyzing the scores regarding the age of the students, we did not find any correlation. A possible explanation is that, as some authors state, the intention to use digital technologies for learning depends more on information literacy than on the age of the participants [40]. Thus, the acceptance of technology for learning in collaborative groups among nursing students does not depend on age.

### 4.5. Limitations and Strengths

This study has some limitations. The main one is that our sample is convenience sampling. The participants were nursing students from a single University of Spain. The participation was voluntary, so there is a potential selection bias that can affect the external validity of the conclusions obtained. The median age of the participants was 18.5 years, similar to other similar research [9,39], and most were women. Albeit most nursing students in Spain are women [32], we should not assume that our sample is representative of all nursing students. Women also tend to use more digital technologies for health [33]. Therefore, these aspects should be considered when assessing the external validity of the results obtained. Another limitation is that our questionnaire was conceived in Spanish, so we cannot infer that our conclusions apply to other languages [25]. Another potential limitation is that the questionnaire was developed in the context of the signature of human anatomy. This aspect should be considered when assessing our findings’ external validity. We must also consider the potential ceiling effect of the scale scores. Other aspects of the educational program can be as important as digital technology for effect. Finally, our questionnaire did not have an online version, which could have helped obtain a larger sample. It could be beneficial to validate this questionnaire with students from other Spanish universities for future research. Translation and transcultural adaptation can also be conducted to examine the validity of the questionnaire in different languages and countries. An online version of the questionnaire could help to increase the sample size with students from other universities or governments. For future research, it could be helpful to analyze if the students perceive that the development of digital technology benefits learning, saving them time.

Our research also had some strengths. Our sample consisted of 132 participants, above the figures suggested by other authors [22]. The participation rate was above the figures displayed by other researchers [7], and the attrition rate, measured as the participants that abandoned, was null. Finally, we must also regard that the questionnaire achieved remarkable results in all the validation and reliability studies that validate this instrument. The evaluation of the participants, real nursing students who performed a collaborative project using technology, also adds value to this research.

## 5. Conclusions

Nursing students positively assess the use of digital technology as a collaborative tool, regardless of their age. The use of digital technology as a collaborative tool is perceived as beneficial as the students find it useful, improves their involvement, and allows them to obtain a better knowledge of their partners. These findings can help develop group projects and tools based on technology to train future nursing professionals for their clinical practice. The questionnaire developed is a valid and reliable tool that can be applied in other colleges or translated into other languages.

## Figures and Tables

**Table 1:** Survey technology-based project in nursing.

Item	Question
During the process of preparing the project with your subgroup:	
1	I have learned anatomy
2	I have been involved in the development
3	I have enjoyed
4	It has helped me to get to know my classmates better
5	It has shown me that humor helps me study
6	I think it is easier to learn by working collaboratively
7	I think we have developed something that helps others
With the presentations of the rest of your classmates:	
8	I have learned anatomy
9	I think they have been involved in their presentations
10	I have enjoyed
11	It has helped me to get to know my classmates better
12	I have been shown that humor helps study
13	I think I can also learn from the work of my classmates
14	I think they have developed projects that help others
How do you rate the activity as a whole?	
15	I found it funny
16	I have learned anatomy
17	I think it should be applied to other subjects
18	Overall, the score I give it is
About yourself:	
19	What is your gender? (male/female)
20	How old are you? (years)

**Table 2:** Sex and age of participants.

Sex	Mean Age	Median	S.D. *	*n*	%
Women	21.1	18.0	6.3	108	82
Men	22.6	19.0	8.4	24	18
Total	21.3	18.5	6.8	132	100

* S.D.: Standard Deviation.

**Table 3:** Total explained variance.

Component.	Total	Variance (%)	Accumulated (%)
1	5.208	37.2	37.2
2	1.442	10.3	47.5
3	1.246	8.9	56.4
4	1.049	7.5	63.9
5	0.832	5.9	
6	0.725	5.2	
7	0.644	4.6	
8	0.569	4.1	
9	0.491	3.5	
10	0.433	3.1	
11	0.422	3.0	
12	0.389	2.8	
13	0.292	2.1	
14	0.259	1.8	

Extraction method: principal factor analysis.

**Table 4:** Factor rotation matrix (a).

Item	Usefulness of the Project of the Student’s Group	Involvement of the Partners	Usefulness of Other Students’ Projects	Better Knowledge of Partners
1		0.798		
3		0.619		
4				0.815
7		0.801		
8	0.506			
9			0.635	
10			0.836	
11				0.797
12	0.517			
13	0.667			
14	0.796			
16	0.826			
17	0.773			
18	0.645			

Extraction method: principal factor analysis. Rotation method: varimax rotation with Kaiser standardization. (a) Five-factor model.

**Table 5:** Reliability tests.

Cronbach’s Alpha	0.853
Split-half Method	0.783
Guttman’s lambda test	0.879
Parallel Model	0.855
Strictly Parallel Model	0.850
Intraclass Correlation Coefficient *	0.853

* Confidence interval 95% (0.814–0.887), *p* < 0.001.

**Table 6:** Regression weights, standard errors, critical ratios, and significances.

Item	Domain	Regression Weight	Standard Error	Critical Ratio	Significance
1	Usefulness of the project of the student’s group	1.000			
2		0.925	0.139	6.664	***
3		0.919	0.131	7.013	***
4		1.115	0.152	7.322	***
5		1.140	0.158	7.238	***
6		1.097	0.169	6.485	***
7		0.835	0.130	6.432	***
8	Involvement of the partners	1.000			
9		0.515	0.119	4.320	***
10		0.962	0.169	5.705	***
11	Usefulness of other students’ projects	1.000			
12		0.815	0.171	4.770	***
13	Better knowledge of partners	1.000			
14		1.860	0.642	2.899	0.004

***: significant.

**Table 7:** Confirmatory factor analysis. Model adjustment measures.

Adjustment Measure	Default Mode	Saturated Mode	Independence Model
NFI	0.839	1.000	0.000
RFI	0.793		0.000
IFI	0.937	1.000	0.000
TLI	0.917		0.000
CFI	0.935	1.000	0.000
GFI	0.898	1.000	0.403
AGFI	0.849		0.311
RMSEA	0.064		0.222
LO 90	0.038		0.206
HI 90	0.087		0.237

**Table 8:** Total score of the domains, adjusted by the number of items.

Domain	*n*	Total Score	S.D. *	Items	Adjusted Score	S.D. *
Usefulness of the project of the student’s group	132	32.71	2.90	7	4.67	0.41
Involvement of the partners	132	14.08	1.35	3	4.69	0.45
Usefulness of other students’ projects	132	9.50	0.81	2	4.75	0.40
Better knowledge of partners	132	8.97	1.27	2	4.48	0.63
Global Score	132	65.27	4.80	14	4.67	0.34

* S.D.: Standard deviation.

**Table 9:** Score of the domains, regarding sex.

Domain	Sex	*n*	Mean	S.D. *	*p*-Value **
Usefulness of the project of the student’s group	Men	108	32.93	2.83	0.04
	Women	24	31.70	3.07	
Involvement of the partners	Men	108	14.22	1.02	0.13
	Women	24	13.45	2.22	
Usefulness of other students’ projects	Men	108	9.55	0.72	0.18
	Women	24	9.25	1.07	
Better knowledge of partners	Men	108	9.00	1.25	0.44
	Women	24	8.79	1.35	
Global Score	Men	108	65.72	4.51	0.03
	Women	24	63.20	5.54	

* S.D.: Standard deviation. ** Mann–Whitney U test.

**Table 10:** Score of the domains, regarding age.

Domain	*n*	Correlation Coefficient	Significance *
Usefulness of the project of the student’s group	132	−0.056	0.523
Involvement of the partners	132	−0.064	0.463
Usefulness of other students’ projects	132	0.062	0.483
Better knowledge of partners	132	0.064	0.463
Global Score	132	−0.014	0.877

* Spearman’s rho correlation coefficient.

## Data Availability

The data presented in this study are available on request from the corresponding author.

## Supplementary Materials

The following supporting information can be downloaded at: https://www.mdpi.com/article/10.3390/ijerph192114267/s1.
