# Supplementary material for: Use of Digital Technology as a Collaborative Tool among Nursing Students—Survey Study and Validation
Source: Int J Environ Res Public Health. 2022 Nov 1;19(21):14267. doi: 10.3390/ijerph192114267 (PMC9659179; doi:10.3390/ijerph192114267)
Supplement: Supplementary file 1 [file ijerph-19-14267-s001.zip › ijerph-1939079-supplementary.pdf]

Supplementary Material. Examples of projects developed by the nursing students.

The students' project involved developing a digital instrument that taught anatomy to their partners entertainingly using digital technology as a collaborative instrument. We show two examples of two of the projects developed and presented by the nursing students.

Example 1. Interactive presentation of the Willis Polygon.

In this project, the students developed an interactive presentation using the software PowerPoint, where the students tried to name different structures of the Willis Polygon. The structures were compared to different parts of the drawing of a Princess, and the students even built an actual model that could be manipulated to show the different parts of the princess/ Willis polygon, which served as a mnemonic rule to remember the actual name of the anatomic structures.

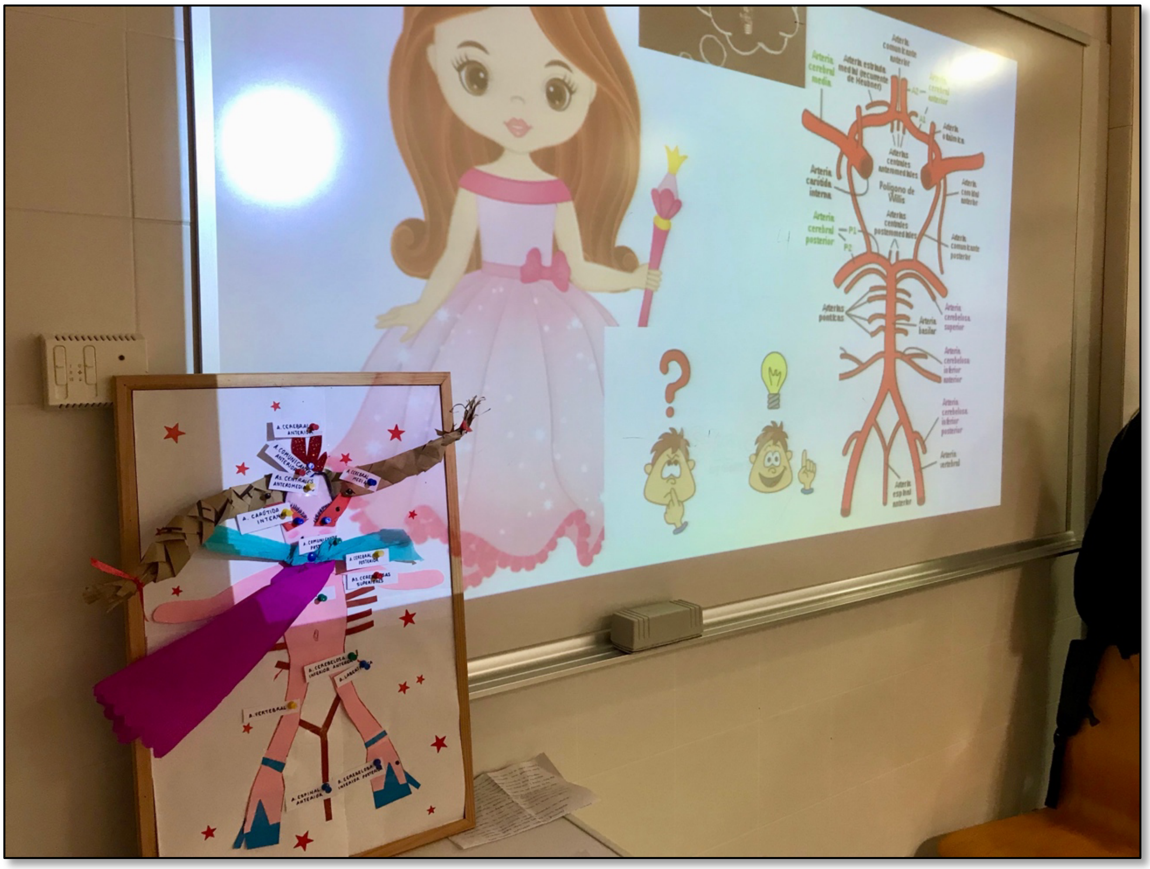

Figure S1. Interactive presentation of the Willis Polygon.

Example 2. Interactive app showing the anatomy of the human heart.

In this project, the students developed an interactive web app in which they had to name structures of the human heart pointed by a virtual finger. Then, the virtual hand put a yellow sticker on the structure to name it, showing if the student had named the proper structure of the anatomy of the human heart.

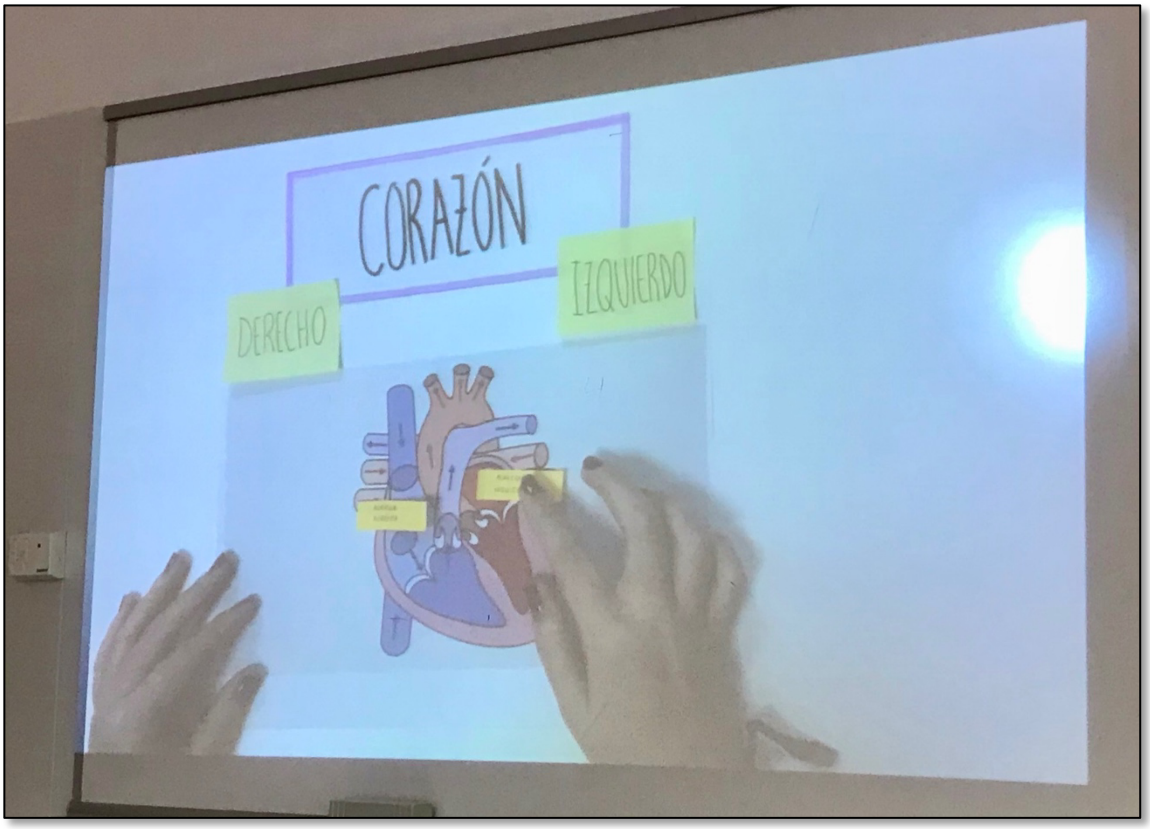

Figure S2. Interactive app showing the anatomy of the human heart.
